# Targeting Insulin Resistance Through Nutrition: Pathophysiological Insights and Dietary Interventions

**DOI:** 10.3390/nu18071119

**Published:** 2026-03-31

**Authors:** Amelia Caretto, Anna Zanardini, Giulio Frontino, Erika Pedone

**Affiliations:** 1 Diabetes Unit, IRCCS San Raffaele Scientific Institute, via Olgettina 60, 20132 Milan, Italy; caretto.amelia@hsr.it (A.C.); zanardini.anna@hsr.it (A.Z.); 2 Department of Pediatrics, IRCCS San Raffaele Scientific Institute, via Olgettina 60, 20132 Milan, Italy; frontino.giulio@hsr.it

**Keywords:** insulin resistance, obesity, Mediterranean diet, metabolic diseases, inflammation, cardiometabolic risk, carbohydrates, nutritional therapy, insulin sensitivity

## Abstract

Background: Insulin resistance (IR) is a hallmark of metabolic disorders including type 2 diabetes mellitus (T2DM), metabolic syndrome, metabolic dysfunction-associated steatotic liver disease (MASLD), obesity, polycystic ovary syndrome (PCOS), and cardiovascular diseases. It arises from impaired insulin signaling in muscle, liver, and adipose tissue, driven by ectopic lipid accumulation, chronic inflammation, oxidative stress, and gut microbiota dysbiosis. Methods: This narrative review synthesizes IR mechanisms and the evidence on specific dietary patterns. A structured search of PubMed/MEDLINE and Embase (up to January 2026) prioritized RCTs, systematic reviews, meta-analyses, and clinical guidelines. Results: IR assessment relies on the hyperinsulinemic–euglycemic clamp as gold standard, with HOMA-IR and QUICKI as validated clinical surrogates. The Mediterranean diet is the most evidence-supported strategy, with consistent HOMA-IR reductions, a 31% decrease in T2DM incidence (PREDIMED-Plus), and demonstrated efficacy across T2DM, MASLD, and PCOS. Low-GI and DASH diets improve postprandial insulin dynamics and are particularly effective in PCOS. Low-carbohydrate and ketogenic diets produce the largest short-term reductions in fasting glucose and insulin secretion, though long-term sustainability requires further study. Plant-based diets and intermittent fasting improve IR primarily via weight loss and gut microbiota modulation. Most studies rely on surrogate IR indices and are short-term (≤26 weeks). Conclusions: Dietary pattern selection should be individualized according to metabolic phenotype, comorbidities, and adherence potential. Larger, longer, head-to-head trials measuring hard clinical outcomes are needed.

## 1. Introduction

Insulin resistance (IR) is a distinctive characteristic of metabolic disorders such as type 2 diabetes (T2DM), metabolic syndrome and non-alcoholic fatty liver disease. When insulin-responsive tissues including muscle, liver and adipose tissue fail to respond effectively to insulin, impaired glucose uptake and systemic metabolic imbalance ensue, leading to IR [1]. IR often develops long before hyperglycemia becomes clinically apparent, driving compensatory hyperinsulinemia and beta-cell dysfunction [2].

Insulin suppresses hepatic glucose production, stimulates glucose uptake in muscle and adipose tissue, and inhibits lipolysis. Disruption of this signaling pathway, particularly via impaired IRS-Akt activity, contributes to insulin resistance [3]. Multiple interrelated factors drive this process: ectopic lipid accumulation in liver and muscle, activation of stress-sensitive kinases due to chronic inflammation and oxidative stress and endoplasmic reticulum stress, all of which impair insulin signaling and glucose homeostasis [4].

IR is frequently present in several pathological conditions and contributes to cardiovascular risk beyond the effects of glycemic control. For these reasons, therapeutic strategies target both glycemic control and underlying metabolic dysfunction. Nutritional and lifestyle interventions that reduce lipid overload, enhance fatty acid oxidation and attenuate inflammation are pillars of therapy and allow long-term management [5]. Both randomized controlled trials (RCTs) and observational studies have consistently reported the beneficial role of Mediterranean and high-fiber dietary patterns on glycemic control, insulin sensitivity, and diabetes incidence [6]. Pharmacological agents such as metformin, thiazolidinediones and GLP-1 and GIP/GLP1 receptor agonists improve insulin sensitivity through different mechanisms [1].

Overall, insulin resistance is a multifactorial disorder driven by complex interactions between impaired insulin signaling, lipid accumulation, and cellular stress. A combined approach integrating pharmacological treatment, lifestyle modification and, above all, dietary strategies offers the most effective way to improve insulin sensitivity and limit progression to metabolic disease.

Here, we provide an overview of IR pathogenesis and its clinical implications, and we systematically examine the evidence on the effects of specific dietary patterns, including the Mediterranean diet, low-glycemic index diets, low-carbohydrate/ketogenic diets, plant-based diets, and intermittent fasting, on insulin sensitivity and IR-associated metabolic outcomes.

## 2. Methods

We conducted a narrative review performing a structured search of the PubMed/MEDLINE and Embase databases up to January 2026. The primary search string was “insulin resistance AND (nutritional therapy OR diet)”. Additional searches used targeted keywords: “obesity OR MASLD OR PCOS OR metabolic disease OR cardiovascular risk” combined with “nutritional management OR dietary pattern OR mediterranean diet OR low glycemic index diets OR plant-based diets OR low-carbohydrate diets OR nutritional guidelines”. Studies were included if they: (i) were published in English; (ii) investigated dietary or nutritional interventions for insulin resistance in adult populations; (iii) reported outcomes related to insulin sensitivity or resistance (e.g., HOMA-IR, fasting insulin, hyperinsulinemic–euglycemic clamp); and (iv) belonged to the following study designs: randomized controlled trials (RCTs), systematic reviews, meta-analyses, real-world evidence studies, or clinical guidelines. Studies were excluded if they: (i) focused exclusively on pharmacological interventions without a dietary component; (ii) were conference abstracts without peer-reviewed full-text data; or (iii) were not published in English. As this is a narrative review, no formal quality appraisal scoring tool (e.g., ROBINS-I, Cochrane Risk of Bias) was applied. Evidence was qualitatively assessed based on methodological rigor (randomization, blinding, presence of control groups), sample size adequacy, validity of IR measurement tools, intervention duration, and clinical relevance of reported outcomes. Limitations and potential sources of bias are explicitly discussed within each dietary section and in the Discussion. The reporting of this narrative review was guided by the SANRA (Scale for the Assessment of Narrative Review Articles) framework to ensure structural consistency and scientific rigor.

## 3. Prevalence and Costs of Insulin Resistance

IR is closely related to serious chronic conditions, such as obesity, T2DM and cardiovascular diseases, that are becoming increasingly prevalent worldwide; consequently, IR indirectly contributes to substantial mortality, morbidity, and economic strain [7]. It is estimated that IR and metabolic syndrome affect approximately one in four to five individuals [8]. Beyond physical and psychological burden, these conditions weigh heavily on healthcare systems and increase vulnerability to infectious diseases, as seen during the COVID-19 pandemic [9].

Metabolic diseases and IR are closely linked through shared molecular and immune mechanisms, leading many individuals to develop multiple coexisting conditions that negatively affect each other. For example, among patients with T2DM, the global prevalence of MASLD is 55.5% (95% CI 47.3–63.7) and up to 50% of hypertensive patients have MASLD [10]. Because these disorders often share common pathways, targeting IR, through precision-based and sustainable nutritional approaches, could allow simultaneous treatment of multiple metabolic diseases, improving patient outcomes while reducing healthcare costs.

## 4. How to Assess Insulin Resistance

A valid diagnostic test for insulin resistance should quantitatively reflect the biological response to endogenous or exogenous insulin in the context of ambient glycemia. Quantitative assessment of insulin resistance is therefore useful for identifying its presence and severity, especially in individuals without established abnormalities in glucose tolerance or diabetes. Despite its clinical relevance, insulin resistance is rarely quantified in routine clinical settings and remains predominantly a research tool. Methods for evaluating insulin resistance were categorized into three main groups: dynamic tests, surrogate indices and biochemical markers [11]. For the diagnosis of IR, the hyperinsulinemic–euglycemic clamp test (HEC) is considered the gold standard, but its clinical applicability is limited due to its complexity and cost [11]. An alternative approach is the frequently sampled intravenous glucose tolerance test (FSIVGTT), which offers detailed assessment of both insulin sensitivity and pancreatic beta-cell function. This method involves intravenous administration of glucose followed by frequent measurements of glucose and insulin, allowing mathematical modeling of insulin dynamics [12]. Like the hyperinsulinemic–euglycemic clamp, it is demanding and costly, and is commonly used in tertiary centers.

Several less invasive surrogate measures are available to estimate IR, including the quantitative insulin sensitivity test index (QUICKI), homeostatic model assessment (HOMA), Matsuda, fasting insulin test, insulin release test and oral glucose tolerance test (OGTT) [13]. These indices are widely applied in epidemiological and clinical research to estimate the risk of diabetes onset in non-diabetic populations. However, their implementation in routine clinical practice is limited by the lack of well-established reference ranges defining normal and impaired insulin sensitivity. Among the available surrogate measures, HOMA-IR, QUICKI and the Matsuda index appear more suitable for clinical use, whereas the HES, McAuley, Belfiore, Cederholm, Avignon and Stumvoll indices are primarily employed in epidemiological or research settings [14]. In addition, HOMA-IR and QUICKI offer practical, cost-effective alternatives for screening and large-scale epidemiological studies, albeit with less precision. The OGTT provides intermediate utility, especially when dynamic glucose–insulin interaction data are desired.

In Table 1, we summarize the main diagnostic techniques and biomarkers for insulin resistance, specifying the procedure for each method and its respective fields of applicability based on clinical context, balancing accuracy, invasiveness, cost and feasibility.

## 5. Insulin Resistance and Metabolic Diseases: A Close Interaction

As insulin is the main hormone that regulates blood sugar, IR is a central component of diabetes mellitus, from prediabetes to complications, through impaired beta-cell function and disrupted glucose metabolism. Although type 1 diabetes (T1DM) mainly results from autoimmune beta-cell destruction and absolute insulin deficiency, increasing evidence shows that adolescents and adults with T1DM frequently develop IR, particularly in the liver, muscle, and adipose tissue [15]. Long-term insulin therapy may further promote IR, leading many T1DM patients to develop features representative of T2DM, including cardiovascular disease and obesity that is becoming increasingly common among individuals with T1DM [16]. T2DM is characterized by progressive beta-cell dysfunction in the setting of IR, genetic susceptibility and environmental factors such as chronic overfeeding. This combination drives a detrimental loop of hyperinsulinemia, lipotoxicity, and glucotoxicity, ultimately resulting in beta-cell failure [2]. Obesity, especially excess visceral and hepatic fat, plays a key role in this process, as IR at the beta-cell and adipose tissue level increases lipolysis and circulating free fatty acids, further impairing insulin secretion and action [2]. Studies using the euglycemic–hyperinsulinemic clamp have demonstrated that both normal-weight individuals with T2DM and non-diabetic subjects with obesity exhibit severe IR, with glucose uptake reduced by approximately 40% compared with healthy controls [8]. This common metabolic defect explains the clinical observation that weight gain in lean individuals with diabetes leads to worsening glycemic control. Controlled feeding studies [17] confirm that negative energy balance reduces hepatic fat and enhances insulin responsiveness and show that a negative energy balance remains a primary therapeutic driver of IR improvement.

Despite comparable degrees of IR, due to impairment of the glycogen synthesis pathway and an abnormal intracellular glucose utilization, individuals with obesity and patients with T2DM differ markedly in glucose tolerance. Subjects with obesity are able to maintain normoglycemia through a substantial compensatory increase in pancreatic beta-cell insulin secretion, resulting in hyperinsulinemia. In contrast, individuals with T2DM exhibit a relative defect in insulin secretion, insufficient to fully overcome IR, leading to impaired glucose tolerance [2]. The link between obesity, chronic inflammation and insulin resistance is central and supported by well-characterized molecular mechanisms [18]. Obesity is increasingly recognized not merely as a matter of excess fat, but as a state of chronic, low-grade inflammation that significantly contributes to the development of insulin resistance. Expansion of white adipose tissue (WAT) in obesity leads to metabolic stress within adipocytes, triggering the release of pro-inflammatory cytokines such as TNF-α, IL-6, IL-1β, and chemokines like MCP-1, creating a self-perpetuating cycle that disrupts normal insulin signaling [19].

As mentioned above, beyond the adipose tissue, obesity-induced inflammation affects metabolically active organs such as the liver and skeletal muscle, promoting steatosis and impairing insulin-mediated glucose metabolism [20].

A group of individuals with obesity, referred to as metabolically healthy obese (MHO), exhibit preserved insulin sensitivity, smaller visceral adipocytes, lower visceral fat, favorable lipid profiles and higher fitness levels despite elevated body mass index (BMI), indicating that obesity does not uniformly confer metabolic risk [21].

While compensatory hyperinsulinemia plays a protective role by limiting the harmful effects of hyperglycemia on vulnerable tissues such as the retina, peripheral nerves and kidneys, chronic insulin resistance and hyperinsulinemia are themselves associated with adverse metabolic and vascular consequences. These include hypertension, dyslipidemia and direct pro-atherogenic effects on the vascular system, which collectively add to the increased cardiovascular risk observed in obesity, impaired glucose tolerance and T2DM [22].

Long-term epidemiological evidence, including the 12-year San Antonio Heart Study, together with insulin clamp studies, demonstrates a strong association between hyperinsulinemia and hypertension [23].These results indicate that many normal-weight individuals with high blood pressure are severely insulin resistant, primarily due to defects in insulin-stimulated muscle glucose uptake and glycogen synthesis, and that approximately half of patients with essential hypertension meet criteria for the IR syndrome [23]. Thus, each element of the IR syndrome represents an important risk factor for cardiovascular disease and, taken together with obesity, hypertension and dyslipidemia, accelerate the development of coronary artery disease (CAD), as demonstrated by findings from the Prospective Coronary Artery Münster (PROCAM) study, which followed 2754 middle-aged men over a four-year period [24,25].

Beyond glycemic dysregulation, IR is independently linked to both macrovascular and microvascular diabetic complications. Clinical markers such as the triglyceride–glucose (TyG) index demonstrate strong associations between IR and vascular damage, renal injury, and arterial stiffness [26,27].

IR is a major contributor to the development of cardiovascular and cerebrovascular diseases across both diabetic and non-diabetic populations [28]. Because the heart is a highly insulin-sensitive organ with high energy demands, experimental studies show that IR alters key insulin signaling pathways, such as IRS1 and IRS2, leading to defective cardiac energy regulation, activation of stress-related kinases and progressive cardiac dysfunction [29,30]. IR is strongly associated with structural and functional vascular abnormalities, including increased arterial stiffness, elevated pulse wave velocity, and impaired myocardial strain with sex-specific differences [31,32]. Elevated circulating fatty acids and dyslipidemia commonly seen in IR further contribute to cardiomyopathy, as diabetic cardiomyopathy, which is characterized by myocardial lipid accumulation, diastolic dysfunction, and left ventricular hypertrophy even in the absence of overt coronary disease [33,34].

The liver plays a key role in maintaining metabolic homeostasis, and insulin resistance (IR) has a bidirectional relationship with MASLD. Excess lipid accumulation in hepatocytes not only characterizes MASLD but also contributes to systemic metabolic dysregulation through signaling components such as lipoproteins, ketones, acylcarnitines, and bile acids [35]. Hyperinsulinemia promotes hepatic lipogenesis both directly and indirectly by increasing circulating free fatty acids and impairing insulin-mediated suppression of glucose production. IR is a key predictor of MASLD across both lean and obese populations and is closely associated with hepatic inflammation, steatosis severity, and histological progression [36]. Moreover, MASLD markedly elevates the risk of type 2 diabetes, particularly in patients with non-alcoholic steatohepatitis, and is linked to hepatocellular injury, pro-inflammatory cytokine activity, dyslipidemia, and fibrosis, highlighting the intertwined pathophysiology of IR and liver disease [37]. Low-carbohydrate diets have potential mechanisms that include reduced hepatic de novo lipogenesis, decreased insulin demand, and a reduction in postprandial glucose excursions and circulating insulin levels [38].

## 6. Insulin Resistance and Other Conditions

Substantial evidence identifies IR and compensatory hyperinsulinemia as central contributors to polycystic ovary syndrome (PCOS) and reproductive impairment, regardless of obesity status, and highlights its central role in both the endocrine and metabolic features of the syndrome [39]. In this condition, hyperinsulinemia exacerbates androgen excess through multiple mechanisms: it suppresses hepatic sex hormone-binding globulin production, increases circulating free testosterone and stimulates androgen synthesis via pituitary, ovarian and adrenal pathways [40]. Insulin also directly enhances ovarian androgen production by activating key steroidogenic enzymes in theca cells and amplifying insulin-like growth factor-1 signaling within the ovary [40].

Growing evidence indicates that IR may represent an important target for cancer prevention, given its involvement in the development of several types of cancers, including breast, colorectal, pancreatic, prostate, endometrial, and adrenal tumors [41], even in individuals without diabetes. In a large observational study, breast cancer incidence in women with high HOMA-IR is associated with all-cause mortality, especially in postmenopausal women [42]. Although the exact mechanisms differ among cancer types, insulin dysregulation (mediated by INSRs, IGF1Rs and INSR/IGF1R hybrids), chronic hyperinsulinemia, inflammation, altered growth factor pathways (non-coding RNAs and mitogen-activated protein kinase (MAPK) insulin pathway) and visceral adipose dysfunction collectively create a pro-tumorigenic environment [43].

Thus, the pathogenic role of IR involves endocrine dysregulation, chronic inflammation, alterations in tissue composition and gut microbiota changes, highlighting its pervasive impact across multiple organ systems [44].

## 7. Mechanisms Underlying Insulin Resistance: Insulin Receptors and the Environment

At the molecular level, IR arises from defects in insulin receptor function, impaired intracellular signaling, disturbances in the cellular microenvironment (inflammation, hypoxia, lipotoxicity), organelle dysfunction, and gut microbiota alterations [45].

Under physiological conditions, insulin binds to the insulin receptor tyrosine kinase (IRTK) on target cells, activating downstream signaling pathways such as IRS-PI3K-Akt, which promotes glucose uptake via GLUT4 translocation in muscle and adipose tissue, suppression of hepatic gluconeogenesis, and enhancement of glycogen and lipid synthesis [46]. In IR, defects at any point in this cascade impair these metabolic actions and contribute to metabolic dysregulation, while chronic inflammation, mediated by cytokines and immune cell infiltration, and gut microbiota dysbiosis further contribute to IR progression [47,48].

Dietary exposures are major modifiable determinants of the mechanisms underlying IR, such as myocellular and hepatic lipid accumulation (diacylglycerols, ceramides), impaired insulin signaling (IRS-1/PI3K/Akt pathway disruption), chronic low-grade inflammation (TNF-α, IL-6, CRP), and oxidative stress and mitochondrial dysfunction [49,50].

**Figure 1:**
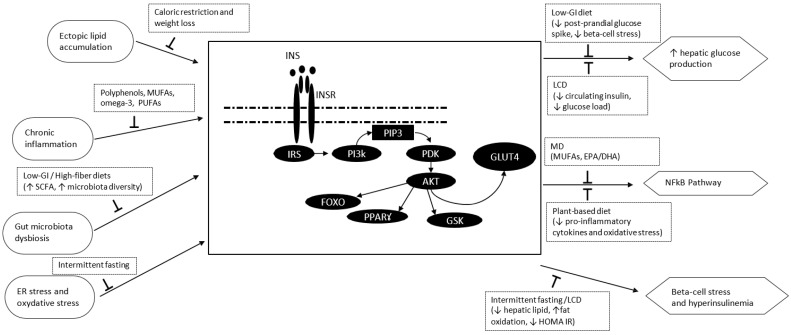
Schematic overview of the molecular pathways underlying insulin resistance (IR) and the principal targets of dietary interventions. The central column illustrates the insulin signaling cascade from the insulin receptor (INSR/IRTK) through IRS-1/IRS-2, PI3K → PIP3, and Akt/PKB to GLUT4-mediated glucose uptake. The left column shows four main pathological drivers of IR. The right column depicts downstream metabolic consequences of impaired signaling. Black arrows represent the mechanistic actions of specific dietary bioactives and patterns referenced in the text: the Mediterranean diet exerts anti-inflammatory effects via polyphenols (oleuropein, resveratrol), MUFAs, and omega-3 PUFAs (EPA/DHA), suppressing NF-κB, JNK and IKKβ activity (Section 8.3 and Section 9); low-GI/high-fiber diets promote SCFA production (Section 8.5); ketogenic and low-carbohydrate diets reduce DAG/ceramide accumulation via β-oxidation and lower circulating insulin (Section 8.7); plant-based diets modulate gut microbiota composition and reduce pro-inflammatory cytokines (Section 8.6); and intermittent fasting induces autophagy, reduces ER stress and ROS, and improves mitochondrial function (Section 8.8). SCFA: short-chain fatty acid; IRS: insulin receptor substrate; PI3K: phosphoinositide-3-kinase; Akt: protein kinase B; GLUT4: glucose transporter type 4; NF-κB: nuclear factor κB; GSK: glycogen synthase kinase-3β; HOMA-IR: homeostatic model assessment of insulin resistance; MD: Mediterranean diet; ER: endoplasmic reticulum; Low-GI: low-glycemic; LCD: low-carbohydrate diet.

The mechanistic framework presented above provides the biological rationale for the dietary interventions discussed in the following sections. Specifically (Figure 1): (i) caloric restriction and weight loss reduce ectopic lipid accumulation (diacylglycerols, ceramides) in liver and skeletal muscle, thereby restoring proximal insulin signaling through the IRS-1/PI3K/Akt pathway; (ii) fiber-rich, low-GI dietary patterns modulate gut microbiota composition, promoting short-chain fatty acid (SCFA) production that activates GPR41/43 receptors and AMPK-mediated insulin sensitization, while simultaneously attenuating TLR4-driven inflammatory signaling; (iii) the anti-inflammatory bioactives characteristic of Mediterranean and plant-based diets (polyphenols, MUFAs, omega-3 PUFAs) suppress IKKβ/JNK and NF-κB activation, reducing cytokine-driven serine phosphorylation of IRS-1 that impairs insulin receptor substrate function; and (iv) ketogenic diets shift energy metabolism toward fatty acid oxidation and ketone body production, reducing glucose-driven insulin secretion and potentially modulating PPAR-γ activity. However, the independence of these effects from concurrent weight loss remains to be established. Nutritional strategies targeting these mechanisms are discussed in detail in Section 8.

In Table 2, we summarize the main mechanisms underlying insulin resistance.

## 8. Management of Insulin Resistance

As previously mentioned, IR is strongly associated with modern lifestyle factors, including high-calorie diets, sedentary behavior, irregular eating patterns, late-night snacking and chronic stress. These factors jointly exacerbate metabolic stress and contribute to the pathogenesis of T2DM. Evidence consistently supports lifestyle modification as the basis for both prevention and treatment of IR, with pharmacological interventions reserved for more severe situations.

### 8.1. Non-Drug Interventions: Lifestyle Modification, Physical Exercise and Diet to Fight Insulin Resistance

Lifestyle modification is a crucial part of IR management and T2DM prevention, with clinical guidelines from major diabetes societies recommending structured lifestyle interventions, including weight loss (typically 7–10% of body weight), regular physical activity (at least 150 min per week of moderate intensity exercise) and behavioral support, as first line therapy for improving insulin sensitivity and reducing diabetes risk [51]. Regular exercise enhances insulin sensitivity and glucose control by means of multiple pathways, such as increasing mitochondrial function and oxidative capacity in skeletal muscle and adipose tissue, improving insulin signaling pathways (including GLUT4, IRS 1/Akt), and reducing pro-inflammatory adipokines and circulating lipids [52].

Physical activity also exerts anti-inflammatory and metabolic effects that counteract the pathophysiology of IR: it enhances mitochondrial biogenesis and function, attenuates signaling pathways associated with inflammation and oxidative stress, and modulates nutrient handling in peripheral tissues [53]. Furthermore, both aerobic and resistance training improve insulin sensitivity and metabolic health, showing synergistic advantages in combined modalities; even non-traditional forms of movement such as yoga and tai chi have been associated with improvements in cardiometabolic markers, reduced waist circumference, and enhanced insulin sensitivity in people with metabolic syndrome or prediabetes [54].

### 8.2. Macronutrients and Dietary Strategies for Insulin Resistance

Diet is central in the management of insulin resistance (IR) and associated metabolic diseases. Caloric restriction, weight loss and intermittent fasting have been shown to ameliorate glycemic control, enhance insulin sensitivity and reduce chronic low-grade inflammation [55]. Even modest reductions in body weight can significantly improve HOMA-IR and fasting glucose.

Carbohydrates (CHOs) are the main driving force for glucose peaks after meals and consequently for insulin secretion, so they may be considered the primary driver of glucose impairment. However, there is no clear correlation between daily amount of carbohydrates and IR. Many studies support the hypothesis that high-carbohydrate low-fat diets may improve glucose control and insulin sensitivity [56,57]. On the contrary, studies about low-CHO dietary patterns support the hypothesis that a lower quantity of CHO may lead to a decrease in insulin postprandial peak and an improvement in insulin sensitivity [58]. It is worth noting that this type of effect may be a direct consequence of weight loss and not only of daily quantities of CHO. Within this framework, the quality of CHO more than the quantity should be taken into account. CHOs may differ in terms of glycemic index (GI) (an index of how fast blood glucose rises after their consumption) and glycemic load (GL) (derived from carbohydrate amount and glycemic index) [59]. The type and timing of carbohydrate intake may also influence insulin dynamics. Low glycemic index (GI) and fiber-rich foods, such as whole grains and legumes, attenuate postprandial glucose spikes, reduce β-cell stress and modulate inflammatory signaling pathways in adipose tissue [60]. Eating more carbohydrates at lunch rather than breakfast or dinner further improves postprandial glucose and insulin responses, likely by coordinating nutrient intake with circadian insulin secretion patterns [61].

The effect of protein intake on IR has been studied, but the literature reports conflicting results. High consumption of animal protein is associated with high risk of developing T2DM and IR, regardless of BMI [62]. In contrast, plant-based protein appears to improve IR [63]. High-protein dietary patterns have been associated with upregulation of the mTOR/S6K9 pathway, with an increase in glucagon levels, with a high glycogen turnover and with increased gluconeogenesis [64]. Therefore, as with carbohydrates, the quality of protein intake is as important as the quantity. Moreover, it is difficult to distinguish the direct effect of protein intake per se from the effect mediated by a consequent increase in lean mass.

Dairy consumption, particularly low-fat products, may enhance insulin sensitivity and reduce visceral adiposity, potentially via calcium and vitamin D-mediated effects on adipocyte metabolism and inflammation [65,66].

Specific dietary patterns confer additional benefits beyond caloric restriction. Mediterranean diet (MD), rich in fruits, vegetables, whole grains, legumes and healthy fats, as well as moderate intake of fish and poultry, boost insulin sensitivity, reduce HOMA-IR and promote satiety [67]. High-protein diets, particularly plant-based, can enhance energy expenditure, preserve lean mass and improve postprandial glucose control, collectively supporting better insulin action.

In the following sections, we discuss different dietary patterns and their effects on IR; the evidence is summarized in Table 3. For each dietary pattern, we report: (i) definition and key nutritional characteristics; (ii) evidence from published papers on IR outcomes (primarily HOMA-IR and fasting insulin); (iii) mechanistic pathways linking the dietary pattern to improved insulin signaling (cross-referenced to Section 7 and Figure 1); (iv) relevant evidence from specific IR-associated conditions (T2DM, MASLD, PCOS, obesity) where available; and (v) limitations including intervention duration, adherence, and weight-loss confounding.

### 8.3. Mediterranean Diet and Insulin Resistance

MD is a traditional dietary pattern rooted in the cultural and socioeconomic context of Mediterranean countries. It is recognized as a diet that incorporates traditional recipes and sustainable lifestyles and has been associated with improvements in metabolic parameters. According to the recent expert consensus on MD, this dietary pattern is characterized by: (i) a high intake of vegetables, fruits, legumes, whole grains, and nuts; (ii) the use of extra virgin olive oil as the main source of dietary fat; (iii) a moderate consumption of fish, seafood, poultry, eggs, milk, and dairy products; (iv) a limited intake of red and processed meats; (v) an occasional consumption of sweets and ultra-processed foods; (vi) elimination of alcohol [68]. Greater adherence to the MD helps ameliorate metabolic diseases such as T2DM, metabolic syndrome, dyslipidemia, MASLD and obesity as it improves several metabolic markers: body mass index (BMI), waist circumference (WC), fat mass, fasting glucose, glycated hemoglobin (HbA1c), Low-Density Lipoprotein Cholesterol, triglycerides, *C*-reactive protein [68,69].

High adherence to the MD directly influences insulin sensitivity and is inversely related to the HOMA index [70], an effect that appears to be attributable in particular to high fish consumption. The MD appears to be beneficial across several conditions associated with IR, such as T2DM, obesity, PCOS and MASLD [71]. This dietary pattern provides a high content of monounsaturated fatty acids (MUFA), dietary fibers, polyphenols, and antioxidant compounds, which are thought to jointly contribute to improved insulin sensitivity.

The positive effect of MD seems to be present regardless of sex and age, with demonstrated effectiveness even in younger populations [72]. A randomized controlled trial (RCT) on 70 female adolescents, randomized to a prescribed MD or generic dietary advice, found that the MD group showed improvements in several metabolic markers, especially WC, body weight and BMI, systolic blood pressure, blood glucose and lipid values, and HOMA index [73]. An RCT conducted in postmenopausal women with obesity showed that the MD was successful in achieving a reduction in insulin levels and HOMA index values [74]. In children with prediabetes as well, the MD was effective in reducing HbA1c and insulin levels [75].

In the PREDIMED trial, a Mediterranean diet supplemented with extra-virgin olive oil or nuts significantly reduced the incidence of major cardiovascular events compared with a low-fat control diet, independent of changes in body weight [76]. Secondary analyses from the same cohort reported reductions in fasting glucose, fasting insulin, and HOMA-IR, supporting a direct effect on insulin sensitivity and risk of T2DM [77].

Additional RCTs have shown that the MD improves postprandial glucose and insulin responses compared with low-fat or Western dietary patterns, alongside reductions in inflammatory markers [78].

Meta-analyses of RCTs further confirm that Mediterranean dietary interventions are associated with significant improvements in HOMA-IR and HbA1c, with effects sustained over long-term follow-up [79]. Mechanistically, these benefits have been attributed to favorable changes in lipid profiles, reduced chronic inflammation, improved endothelial function, and modulation of gut microbiota composition [80].

The PREDIMED-Plus trial (2025) showed that a low-calorie Mediterranean diet combined with moderate physical activity and professional support reduced T2DM risk by 31% in overweight or obese adults with metabolic syndrome, compared with a standard Mediterranean diet. This intervention also promoted greater reductions in weight, waist circumference, and fasting insulin, highlighting the additive effects of lifestyle modifications on insulin resistance [81]. The positive effect of the MD should be interpreted as a comprehensive effect encompassing weight loss and improvement in multiple metabolic parameters as showed in Table 4.

### 8.4. DASH Diet and Insulin Resistance

DASH (Dietary Approaches to Stop Hypertension) is a dietary plan that recommends eating vegetables, fruits, and whole grains; including fat-free or low-fat dairy products, fish, poultry, beans, nuts, and vegetable oils; limiting foods that are high in saturated fat, such as fatty meats, full-fat dairy products, and tropical oils such as coconut, palm kernel, and palm oils; limiting sugar-sweetened beverages and sweets [82].

This dietary pattern has been proven effective in reducing IR [83]. In particular, in a population of women with IR and PCOS, the DASH diet was shown to reduce insulin levels and HOMA index values. Foroozanfard and colleagues [84] randomized a cohort of women with PCOS to hypocaloric control diet or hypocaloric DASH diet and observed a reduction in fasting insulin levels, HOMA index values and an increase in insulin sensitivity. The nutritional plans included 52–55% carbohydrates, 16–18% proteins and 30% total fats; but the DASH diet was rich in fruits, vegetables, whole grains, low-fat dairy products, cholesterol and refined grains. Asemi and colleagues [85] obtained similar results in a population of women with PCOS using a DASH plan in which total daily energy was 18% from protein, 52% from carbohydrates, 30% from fats, and was low in sodium. Table 5 summarizes the effect of the DASH diet on different populations and a comparison of the effects on IR of the DASH diet versus the MD that should be further investigated [86].

### 8.5. Low-Glycemic Index Diets and Insulin Resistance

Low-glycemic index (low-GI) diets emphasize carbohydrate quality, favoring foods that induce lower and slower postprandial glucose and insulin responses. This approach emphasizes carbohydrate sources that produce slower and lower postprandial blood glucose responses compared with high-GI foods [87]. This dietary approach has been proposed as a strategy to reduce insulin demand and increase insulin sensitivity, particularly in individuals with impaired glucose regulation.

A recent meta-analysis conducted by Yu et al., including six randomized controlled trials with 192 adult participants without diabetes, demonstrated that low-GI diets significantly reduced HOMA-IR compared with high-GI diets (estimate 0.31; 95% CI 0.01–0.61; *p* < 0.001) [88]. This finding suggests that early intervention and even modest reductions in dietary GI can improve insulin sensitivity in non-diabetic populations.

In an RCT, twenty-two prediabetic adults with obesity underwent a 12-week exercise training intervention (1 h/day for 5 days/week at 85% of maximum heart rate) while randomly assigned to receive either a low-GI diet (GI 40 ± 0.3 units) or a high-GI diet (GI 80 ± 0.6 units). Both groups lost equal amounts of body weight (−8.8 + 0.9%) and adiposity, tested by using dual-energy X-ray absorptiometry and computed tomography, and showed similar improvements in peripheral tissue (+76.2 and 14.9%) and hepatic insulin sensitivity (+27.1 + 7.1%) (all *p* < 0.05). However, oral glucose-induced insulin secretion was reduced only in the low-GI group (6.59 + 0.86 nmol prior to the study compared with 4.70 + 0.67 nmol after the study, *p* < 0.05) [89] (Table 4). These were shown to be independent of the ≈9% decrease in body weight and fat mass but were related to changes in the gastrointestinal incretin peptide, GIP, the postprandial response of which was decreased only in subjects who consumed a low-GI diet. The consumption of a high-GI diet impaired pancreatic β-cell function and prevented the improvement in intestinal K cell function. In contrast, a low-GI diet decreased both hyperglycemia and hyperinsulinemia and thus relieved β-cell stress. These findings highlight the important role of the gut in mediating the effects of a low-GI diet on type 2 diabetes risk reduction.

In pediatric populations, a systematic review and meta-analysis of randomized controlled trials in adolescents and children with overweight/obesity suggested that carbohydrate-modified diets, including low-GI approaches, can improve HOMA IR when implemented alongside calorie restriction or balanced macronutrient distribution (46–65% energy from carbohydrates), although effects on fasting insulin and HOMA IR were heterogeneous across trials [90].

Observational evidence further supports the relationship between habitual dietary low GI and metabolic health. Cohort studies, including the Framingham Offspring Study [91], have demonstrated associations between higher dietary GI and increased insulin resistance, as measured by HOMA-IR, as well as a greater prevalence of metabolic syndrome, consequently reinforcing the rationale for low-GI dietary strategies.

In the Framingham Offspring cohort, 2875 non-diabetic participants were assessed for dietary habits and classified into four dietary patterns: “Fruits, Reduced-Fat Dairy, and Whole Grains,” “Refined Grains and Sweets,” “Beer,” and “Soda.” Compared with individuals adhering to the “Fruits, Reduced-Fat Dairy, and Whole Grains” pattern, those in the “Soda” pattern exhibited significantly higher fasting insulin concentrations. This pattern was also characterized by lower dietary fiber intake and a higher dietary glycemic index, consistent with previous findings linking sugar-sweetened beverage consumption to adverse metabolic outcomes [91].

Overall, low-GI diets represent a practical and evidence-based approach to improving insulin sensitivity across diverse populations, especially when combined with caloric control and high-quality dietary patterns, as reported in Table 6. The metabolic improvements observed may not be attributable strictly to glycemic index. Increased dietary fiber intake, reduced saturated fat consumption, lower energy density, modulation of gut microbiota, and weight loss likely act as mediating or synergistic mechanisms. This is consistent with the growing emphasis on dietary patterns rather than isolated nutrient metrics. However, heterogeneity in study design, population characteristics, and intervention context underscores the importance of personalized nutrition approaches tailored to individual metabolic profiles.

### 8.6. Plant-Based Diets and Insulin Resistance

Plant-based dietary patterns, including vegetarian and vegan diets, are characterized by high intakes of fiber-rich plant foods and reduced consumption of animal products and saturated fats. These diets have been increasingly studied for their potential to increase insulin sensitivity and reduce the risk of T2DM.

In a systematic review, eight randomized controlled trials including a total of 716 participants evaluated the effects of plant-based dietary interventions on markers of insulin sensitivity. In adults with BMI ≥ 25kg/m^2^, plant-based diets (vegan, ovo-vegetarian, lacto-vegetarian, and lacto-ovo-vegetarian) administered for ≥14 days were associated with a significant reduction in HOMA-IR compared with control diets (mean difference −0.97, 95% CI −1.67 to −0.27; *p* = 0.007) and a significant decrease in fasting insulin concentrations (mean difference −4.13 µU/mL, 95% CI −7.22 to −1.04; *p* = 0.009) [92].

In addition to this meta-analytic evidence, RCTs of plant-based dietary interventions have shown that dietary patterns emphasizing whole grains, legumes, and minimal animal products can improve β-cell function and reduce insulin resistance over periods as short as 16 weeks, with effects on HOMA IR correlated with reductions in BMI and visceral fat. In a 16-week randomized clinical trial, 75 overweight adults without diabetes were assigned to a low-fat plant-based diet (*n* = 38) or a control group with no dietary changes. Compared with controls, the intervention group experienced a significant reduction in insulin resistance, with HOMA IR decreasing by approximately −1.0 units (95% CI −1.2 to −0.8; *p* = 0.004), while no significant change occurred in controls. Meal-stimulated insulin secretion increased markedly in the plant-based group compared with controls (*p* < 0.001). Fasting glucose, insulin, and C peptide concentrations were all significantly reduced in the intervention group (all within-group *p* < 0.01) alongside reductions in total, LDL, and HDL cholesterol. Basal insulin secretion declined significantly (*p* = 0.006), and improvements in beta-cell glucose sensitivity were observed (*p* = 0.01), although between-group differences for glucose sensitivity did not reach statistical significance. Changes in HOMA IR correlated positively with decreases in BMI and visceral fat (r = 0.34, *p* = 0.009; r = 0.42, *p* = 0.001) [93].

Similar effects have been observed in overweight and obese individuals following plant-based dietary interventions without explicit energy restriction (approximately 75% of energy from carbohydrates, 15% protein, and 10% fat), consisting of vegetables, grains, legumes and fruits without animal products or added fats. Vitamin B12 was supplemented (500 μg/d). In a 16-week randomized trial, Kahleova et al. (2018) [93] tested a low-fat vegan diet in overweight adults. The diet improved insulin resistance, with HOMA-IR decreasing by −1.3 (95% CI: −2.2 to −0.3; *p* < 0.001) and predicted insulin sensitivity increasing by 0.9 (95% CI: 0.5–1.2; *p* < 0.001) compared to controls. Participants lost 5.9 kg (*p* < 0.001) on average. In a subset, hepatocellular lipid decreased by 34.4% (*p* = 0.002) and intramyocellular lipid by 10.4% (*p* = 0.03), with both reductions correlating with improvements in insulin sensitivity (r ≈ 0.51, *p* = 0.01). The intervention also enhanced postprandial metabolic responses [94].

In a prospective analysis of 6798 middle-aged and older adults from the Rotterdam Study, higher adherence to a plant-based dietary index (range 0–92) was associated with favorable metabolic outcomes over follow-up. Per 10-unit-higher plant-based diet score, there was a significant reduction in longitudinal insulin resistance (HOMA IR β = −0.09, 95% CI −0.10 to −0.08; adjusted β = −0.05, 95% CI −0.06 to −0.04 after BMI adjustment). Higher scores were also linked to lower incidence of prediabetes (HR = 0.89, 95% CI 0.81–0.98) and type 2 diabetes (HR = 0.82, 95% CI 0.73–0.92), though the association with prediabetes was attenuated and non-significant after BMI adjustment (HR = 0.93, 95% CI 0.85–1.03). The inverse association with type 2 diabetes remained significant after adjusting for BMI (HR = 0.87, 95% CI 0.79–0.99), supporting a protective role of plant-based diets against insulin resistance and T2DM [95].

Several mechanisms could explain the improvements in insulin sensitivity observed. First, diets rich in whole plant foods are typically lower in saturated fats, which may reduce fat accumulation in liver and skeletal muscle, thereby improving insulin signaling at the cellular level. Second, high dietary fiber intake from fruits, vegetables, legumes and whole grains can slow glucose absorption, promote beneficial gut microbiome activity and increase production of SCFAs (short-chain fatty acids), which have anti-inflammatory and insulin-sensitizing effects. Additionally, plant-based diets are often lower in energy density, which can facilitate spontaneous weight loss and reductions in adiposity, both of which independently enhance insulin sensitivity.

However, it remains difficult to separate the specific effects of plant-based foods from those resulting from reduced dietary fat or weight loss, especially in low-fat vegan interventions. Many studies show that participants lose weight and reduce total fat intake simultaneously, making it challenging to determine whether improved insulin sensitivity is driven by plant-based composition itself or by caloric restriction and fat loss. Table 7 summarizes the evidence from the main studies conducted on plant-based diets.

Despite these promising findings, heterogeneity in plant-based diet definitions and adherence across studies requires careful interpretation in attributing benefits solely to plant-based patterns and highlights the need for further controlled studies.

### 8.7. Low-Carbohydrate Diets and Insulin Resistance

Low-carbohydrate diets (LCDs) represent a variety of dietary interventions characterized by a significant reduction in daily carbohydrate intake relative to standard Western diets. While the exact definition varies across studies, LCDs are commonly defined as providing less than 45% of total daily energy from carbohydrates, protein typically constituting 20–30% of energy intake and fats comprising the remaining 30–50% [96]. A ketogenic diet (KD) is typically defined as a diet with very low carbohydrate intake (often <50 g/day or <10% of total calories), moderate protein, and high fat, designed to induce sustained nutritional ketosis. Induced ketosis results from shifting energy metabolism away from glucose toward fatty acid oxidation and ketone body production (β hydroxybutyrate, acetoacetate) [97].

A recent systematic review and dose–response meta-analysis of randomized controlled trials in individuals with T2DM found that reducing carbohydrate intake was linearly associated with improvements in glycemic control and markers of insulin resistance. Specifically, each 10% decrease in dietary carbohydrate corresponded to reductions in HbA1c, fasting glucose, body mass index (BMI), fasting insulin, and HOMA IR, with the most significant changes occurring predominantly during the first six months of the intervention. However, these effects diminished beyond this period [98].

In a meta-analysis of KD interventions in individuals with T2DM, KD resulted in clinically meaningful improvements in fasting glucose and HbA1c levels along with weight loss, lipid metabolism and waist circumference implying broader metabolic benefits. The meta-analysis included 567 participants and assessed the impact of the ketogenic diet by comparing pre- and post-intervention biomarkers of glucose and lipid metabolism, as well as body weight. Fasting blood glucose decreased by an average of 1.29 mmol/L after KD interventions and HbA1c (glycated hemoglobin) was reduced by 1.07% on average, indicating improved long-term blood glucose control [99].

RCTs comparing low-carb and low-fat diets demonstrated greater short-term reductions in fasting insulin and HOMA-IR in the low-carb groups, particularly in individuals with obesity. The landmark study by Foster et al. (2003) [100] showed that participants assigned to a low-carb diet not only experienced significant weight loss but also exhibited improved markers of insulin sensitivity, indicating that low-carb diets may confer metabolic benefits beyond weight reduction. These data suggest that low-carb dietary interventions can be particularly effective in managing obesity-related insulin resistance in the short-term, showing their potential therapeutic role in metabolic health [100].

However, not all evidence unambiguously supports carbohydrate restriction for augmenting insulin sensitivity across all populations. Some observational data suggest that habitual low carbohydrate intake (<45% of energy) in otherwise healthy individuals may correlate with dysregulated glucose homeostasis and elevated HOMA IR, HOMA-β, and *C*-peptide levels, as well as markers of metabolic acidosis, including lower serum bicarbonate and albumin and an increased anion gap. The rise in *C*-peptide also correlated positively with IRS-related inflammatory markers (FGF2, IP-10, IL-6, IL-17A, MDC) and negatively with IL-3. These findings indicate that, in healthy normal-weight individuals, low carbohydrate intake may impair glucose regulation, promote metabolic acidosis, and potentially trigger inflammation via elevated *C*-peptide levels [101].

Some controlled interventions that maintained body weight during KD feeding did not show significant improvements in insulin sensitivity, suggesting that weight loss itself is a major driver of observed metabolic improvements in many studies. This highlights the importance of body weight control in interpreting the effects of KD on insulin resistance [102].

In overweight non-diabetic subjects, it was reported that during ketosis fasting, glucose was not affected, but there was an elevation in postprandial blood glucose concentration. These data suggest a different effect of ketosis on glucose homeostasis in diabetic and non-diabetic individuals [103]. In Table 8, we reported the main outcome data of the ketogenic diet in the different populations.

Systematic analysis shows that ketogenic dietary interventions in T2DM may result in substantial reductions in fasting glucose and HbA1c, markers closely tied to improvements in insulin resistance. However, many studies report concurrent weight loss, making it challenging to dissociate weight-loss-mediated from diet-specific improvements in insulin sensitivity [104].

LCDs and KDs may be valuable nutritional approaches in specific diseases (i.e., T2DM, obesity, epilepsy) but require regular periodic monitoring. A reduction in CHO quantity may be associated with a high-fat dietary pattern [59], so lipid profile should be regularly checked. LCDs and KDs are associated with ketosis so they are not recommended in high-risk populations, such as in T1DM, pregnant or lactating women, renal impairment, or eating disorders [59]. Finally, long-term sustainability of this dietary pattern should be further evaluated [59].

### 8.8. Intermittent Fasting and Insulin Resistance

Intermittent fasting is characterized by repeated intentional interruptions in energy intake or a very-low-calorie diet (500–700 kcal) for 2–4 days a week [105,106]. Various fasting regimens such as intermittent fasting, alternate-day fasting, and time-restricted feeding have demonstrated beneficial effects thought to be mediated through decreased hepatic glucose production, improved insulin sensitivity, enhanced lipid metabolism, and reduced inflammation [106,107,108].

In a recent randomized controlled trial, the addition of a personalized 8–10 h time-restricted eating (TRE) intervention to standard-of-care dietary counseling in adults with metabolic syndrome resulted in modest but statistically significant improvements in glycemic control over a 3-month period. Compared with standard care alone, TRE was associated with a greater reduction in HbA_1_c, alongside significant decreases in body weight, body mass index, and central adiposity, with fat mass accounting for the majority of weight loss. TRE showed greater reductions in HOMA-IR compared with standard care, but these differences were not statistically significant in this study. In addition, other glycemic measures such as fasting insulin and fasting glucose also trended in favor of TRE but were not significantly different between groups [109].

In a randomized clinical trial comparing modified alternate-day fasting (ADF) with conventional calorie restriction (CR) in adults with metabolic syndrome over 8 weeks, ADF produced significantly greater reductions in body weight (*p* = 0.003), waist circumference (*p* = 0.026), systolic blood pressure (*p* = 0.029), and fasting plasma glucose (*p* = 0.009) compared with CR. No significant differences were observed between groups in HOMA-IR (*p* = 0.425) [110].

In a 12-week randomized controlled trial comparing energy-restriction intermittent fasting (IER) with continuous energy restriction (CER) in adults with metabolic syndrome, both dietary interventions produced significant improvements in weight and metabolic biomarkers. Participants in both groups lost approximately 7% of baseline body weight, accompanied by reductions in body mass index and waist circumference (*p* < 0.05 for all). Biochemical measures, including systolic and diastolic blood pressure, total cholesterol, triglycerides, LDL-C, fasting glucose, fasting insulin, HbA1c, and HOMA-IR, also decreased significantly from baseline in both groups (*p* < 0.05), but no statistically significant between-group differences were observed for any biomarker [111].

In the Guo et al. trial, intermittent fasting did significantly improve insulin resistance (HOMA-IR) within the IF group, but when comparing the IF group with the control diet, the between-group difference in HOMA-IR was not statistically significant over the 8-week intervention [112]. In an 8-week randomized trial of adults with metabolic syndrome, a “2-day” intermittent fasting (IF) protocol led to significant within-group improvements in insulin resistance, with HOMA-IR decreasing from 2.60 to 1.98 (*p* = 0.012 vs. baseline). IF also produced significant reductions in body weight, BMI, waist circumference, and fat mass (*p* < 0.01), along with favorable changes in adipokine profiles (leptin ↓, adiponectin ↑; *p* < 0.05), oxidative stress, and endothelial function markers. Moreover, the intervention induced beneficial alterations in gut microbiota composition, including increased abundance of SCFA-producing taxa and decreased plasma LPS levels, which correlated with improvements in cardiometabolic indices. Although, between-group differences in HOMA-IR and fasting insulin were not statistically significant [112].

In a multicenter randomized controlled trial, the effect of a 5-day fasting period followed by 10 weeks of lifestyle modification (modified DASH diet, exercise, mindfulness) on insulin resistance was directly assessed against lifestyle modification alone in patients with metabolic syndrome. Although the co-primary outcome of the HOMA index at week 12 did not differ significantly between groups, exploratory analyses revealed that fasting induced a significant short-term reduction in HOMA-IR immediately after the fasting week (Δ = −0.8; 95% CI −1.7 to −0.1; *p* = 0.046) when compared with the control group, indicating an acute improvement in insulin sensitivity following the fasting intervention. This effect, however, was not maintained at week 12 or week 24, as group differences in HOMA-IR at these later time points were non-significant (*p* > 0.6) despite continued lifestyle modification [113].

A systematic review of relevant clinical trials demonstrated that intermittent fasting (IF) was associated with statistically significant improvements in glucose metabolism, insulin resistance, lipid profile, and anthropometric parameters in patients with impaired glucose and lipid metabolism. Regarding glycemic control, IF significantly reduced fasting plasma glucose (mean difference [MD] ≈ −0.15 mmol/L, *p* < 0.05) and HbA1c levels (MD ≈ −0.08%, *p* < 0.05). In addition, circulating insulin levels were significantly decreased (MD ≈ −13 µU/mL, *p* < 0.01), accompanied by a significant reduction in HOMA-IR (MD ≈ −0.31, *p* < 0.05), indicating improved insulin sensitivity. In addition, the average weight decreased by 2.51 kg (95% CIs: −3.27 to −1.75), whereas the waist circumference reduced by 2.25 cm on average (95% CIs:−3.08 cm to−1.42 cm). BMI reduced by −0.82 kg/m^2^ (95% CIs: −1.34 to−0.30) [114].

Several randomized controlled trials, included in Table 9, assessing intermittent fasting (IF) and energy restriction (ER) have investigated their effects on insulin resistance (HOMA-IR) in patients with metabolic syndrome. In the study by Kunduraci & Ozbek [111], both the intermittent energy restriction (IER) and continuous calorie restriction (CER) groups exhibited significant within-group reductions in HOMA-IR (*p* < 0.05), but between-group differences were not significant, indicating that the pattern of caloric restriction did not confer additional benefits on insulin resistance. Similarly, Parvaresh et al. [110] compared alternate-day fasting (ADF) to calorie restriction and found that HOMA-IR did not significantly differ between the two groups, despite significant reductions in body weight, waist circumference, and fasting glucose in the ADF group. In contrast, Guo et al. [112] reported significant within-group improvement in HOMA-IR in the 2-day intermittent fasting group (*p* = 0.012 vs. baseline), but again, no significant difference was found between the IF and control groups, suggesting that improvements in insulin resistance in IF were not greater than those observed with conventional dietary approaches. Finally, in the TRE study [109], HOMA-IR showed no significant changes after 12 weeks, although there were improvements in trunk fat, body weight, and fasting glucose. Collectively, while all diets (IF, ADF, TRE, IER, CER) demonstrated significant benefits in weight loss and other cardiometabolic markers, insulin resistance, as measured by HOMA-IR, was generally unaffected by the pattern of energy restriction, suggesting that weight loss alone is a primary driver of improvements in insulin sensitivity. Future studies with longer follow-up may help clarify the role of intermittent feeding patterns in sustained insulin resistance improvement.

## 9. Nutritional Modulation of Inflammation to Improve IR

Multiple dietary bioactive compounds have been examined for their capacity to regulate nutrient-responsive inflammatory pathways linked to insulin resistance (IR) in obesity. Curcumin, the main polyphenol found in turmeric, inhibits NF-κB activation and lowers pro-inflammatory cytokine release in vitro, potentially enhancing insulin signaling in adipose tissue and other metabolic organs [115]. Likewise, salicylic acid—a naturally occurring compound in numerous plant-based foods—blocks cyclooxygenase-2 and pro-inflammatory gene expression, though the dietary amounts needed to meaningfully suppress NF-κB are unlikely to be attainable from food sources alone.

Long-chain *n*-3 polyunsaturated fatty acids (PUFAs), including EPA and DHA, deliver anti-inflammatory and insulin-sensitizing benefits by stimulating AMPK, altering PPAR-γ activity, blocking NF-κB through the GPR120 receptor, and competitively disrupting pro-inflammatory eicosanoids from arachidonic acid [116]. Combinations of nutrients might amplify immunomodulatory benefits: supplementation with blends of bioactive agents improved insulin resistance and inflammatory adipokine profiles in overweight adults, irrespective of weight reduction. Antioxidants like vitamins C and E mitigate oxidative stress and dampen inflammatory cascades, whereas phytochemicals such as lycopene prevent the initiation of pro-inflammatory mediators [18]. These nutritional elements could offset obesity-driven inflammation, possibly boosting insulin sensitivity, despite mixed clinical data. Nonetheless, results across studies vary owing to differences in dosing, bioavailability, trial length, and personal genetic or metabolic traits, highlighting the value of tailored nutrition to address inflammation-driven IR [18].

## 10. Nutritional Guidelines

Various consensus statements and guidelines exist regarding the management of diseases linked to IR but, to the best of our knowledge, there is a paucity of clear guidelines on the management of IR, alone or in combination with other diseases. In 2021, nutritional guidelines for the management of insulin resistance were published by a panel of international experts in the field [117]. The authors highlight that weight reduction and caloric restriction are the key components of IR management. Specifically, experts suggest:

- Caloric restriction and portion control.
- Healthy eating with whole or unprocessed food.
- Reduction in sodium intake.
- Avoidance of alcohol consumption.
- Increased use of whole grains and fibers in order to reduce glycemic load and glucose index.
- Preference of a plant-based diet.
- Increased intake of insoluble fibers (14 g/1000 kcal per day).

## 11. Discussion

IR serves as a central driver underlying a spectrum of metabolic, cardiovascular, and systemic disorders, including T2DM, obesity, MASLD, PCOS, and oncogenesis, driven by defective insulin receptor signaling, ectopic lipid accumulation, chronic low-grade inflammation, oxidative stress, endoplasmic reticulum dysfunction, and gut microbiota dysbiosis. Specific dietary patterns targeting these mechanisms, including the Mediterranean diet, low-GI diets, low-carbohydrate/ketogenic diets, plant-based diets, and intermittent fasting, represent evidence-based, non-pharmacological strategies for improving insulin sensitivity. Nutritional therapy can act synergistically with pharmacological therapies and should be individualized according to the patient’s metabolic phenotype, comorbidities, and adherence potential.

Nonetheless, gaps still exist, including limited head-to-head comparisons of dietary regimens, suboptimal long-term adherence data in heterogeneous populations (i.e., across ethnicities, ages, and comorbidities), and underexplored interactions with hereditary predispositions. MD has been proven effective in reducing IR in different populations and with different comorbidities. Low-GI diets represent an evidence-based approach to improving insulin sensitivity throughout diverse populations, especially when combined with caloric control and high-quality dietary patterns. Low-carbohydrate and ketogenic dietary patterns represent potent nutritional strategies able to improve glycemic control and insulin resistance, mainly through reducing carbohydrate load, lowering circulating insulin, enhancing fat oxidation, modifying adipokine profiles, and possibly exerting anti-inflammatory effects.

While multiple dietary patterns demonstrate improvements in IR, predominantly measured as reductions in HOMA-IR, direct quantitative comparisons across interventions are substantially limited by heterogeneity in study design, population, intervention duration, and outcome measurement. Among the reviewed patterns, the Mediterranean diet has the broadest evidence base, with consistent HOMA-IR reductions across multiple RCTs and meta-analyses, improvements in hard endpoints (T2DM incidence), and demonstrated effectiveness across diverse conditions including T2DM, obesity, PCOS, and MASLD. Low-GI dietary approaches show reliable surrogate IR improvements with good feasibility and low risk profile, particularly in populations with postprandial hyperinsulinemia. Low-carbohydrate and ketogenic diets demonstrate the largest short-term reductions in fasting glucose and HbA1c, but effects on direct insulin sensitivity are more variable, attenuate beyond six months [98], and long-term sustainability and safety in specific populations (T1DM, renal impairment, eating disorders) remain concerns. Plant-based and intermittent fasting interventions show comparable HOMA-IR improvements to conventional dietary approaches, but between-group differences versus active comparators are frequently non-significant, limiting conclusions about added metabolic benefit beyond caloric restriction alone. Study conclusions are predominantly based on variable surrogate IR indices rather than hard clinical endpoints (T2DM incidence, CVD events), most interventions are short-term (≤12–26 weeks), and study populations are heterogeneous. Furthermore, the study populations are heterogeneous, often studying IR as a component of specific diseases rather than as a distinct clinical entity, which limits generalizability. The evidence level of studies is variable: high (GRADE A) for MD on HOMA-IR (−0.5, meta-analysis of 12 RCTs); moderate for low-carb (−1.1, *I*^2^ = 65%); and low for long-term sustainability of dietary patterns (KD, intermittent fasting). Conclusions implying sustained metabolic benefit should therefore be interpreted with caution.

Weight loss remains a primary and well-established mediator of improvements in IR status across all dietary patterns reviewed. A 5–10% reduction in body weight consistently correlates with clinically meaningful improvements in surrogate IR indices, including HOMA-IR and fasting insulin. Because most dietary intervention studies are conducted in the context of energy deficit, isolating macronutrient- or bioactive-specific effects from weight-loss-mediated improvements requires isocaloric or weight-stable study designs, which are comparatively rare in the literature. Nonetheless, emerging evidence supports the existence of weight-independent, diet-composition-specific effects on insulin sensitivity. The benefits of low-GI diet on postprandial insulin dynamics have been demonstrated in weight-stable exercise trials, suggesting that carbohydrate quality per se modulates beta-cell stress and inflammatory signaling in adipose tissue independently of caloric restriction. Mediterranean diet polyphenols (e.g., oleuropein, resveratrol, hydroxytyrosol) attenuate IKKβ/JNK activation and downstream NF-κB-mediated suppression of IRS-1 signaling, an effect partially dissociable from weight change, as supported by secondary analyses from the PREDIMED trial showing IR improvements without significant between-group differences in body weight. Ketogenic diets, by contrast, show insulin sensitivity improvements predominantly in the context of concurrent weight loss; weight-stable KD interventions [102] have not consistently demonstrated significant independent effects on insulin sensitivity, suggesting that caloric restriction remains a dominant driver of this dietary pattern. For plant-based and intermittent fasting interventions, between-group differences in HOMA-IR are frequently non-significant when energy intake is matched, further supporting the primacy of weight loss. Stable-weight mechanistic studies are needed to definitively characterize the weight-independent metabolic effects of specific dietary patterns.

Tailored recommendations should be based on the patient’s comorbidities, metabolic phenotype, and adherence potential. The Mediterranean diet represents the most evidence-supported first-line nutritional strategy across a broad range of IR-associated conditions given its established effects on hard cardiovascular and metabolic endpoints, favorable safety profile, and cultural acceptability, particularly in the context of CVD risk reduction and MASLD. Low-GI and DASH dietary approaches are evidence-based options with good feasibility, particularly suitable for PCOS and conditions characterized by pronounced postprandial glycemic excursions. Low-carbohydrate diets may be considered in the short-to-medium term for individuals with T2DM to exploit their potent effects on fasting hyperglycemia, insulin secretion, and weight control; however, their use requires regular monitoring of lipid profiles and safety parameters, and their long-term sustainability remains to be established in larger pragmatic trials. Plant-based dietary patterns and intermittent fasting regimens represent reasonable alternatives that may confer additional benefits through gut microbiota modulation and metabolic flexibility, but clinical decisions should account for individual adherence barriers and nutritional adequacy.

Long-term safety, sustainability, and individualized responses continue to be major areas for future research. Future investigations should focus on large-scale, pragmatic trials evaluating personalized nutrition via precision tools. For example, continuous glucose monitoring shows fasting and postprandial glucose profiles that can be used to tailor macronutrient load at meals.

A multidisciplinary, individualized approach for patients with IR is still the best therapeutic strategy for reducing the impact of this pathological condition and the risk of developing cardiometabolic disease.

## Figures and Tables

**Table 1:** Dynamic tests, surrogate indices and biomarkers to assess insulin resistance/sensitivity for clinical use and epidemiological/research purposes.

Category	Method/Index/Biomarker	Clinical Use	Research/Epidemiology Use	Notes
Reference Methods	HEC (Hyperinsulinemic–Euglycemic Clamp)	Rarely	Commonly	Gold standard for insulin sensitivity but labor-intensive and costly procedure requiring IV infusions
	FSIVGTT (Frequently Sampled IV Glucose Tolerance Test)	Rarely	Commonly	Evaluates insulin–glucose dynamics requiring modeling
Dynamic/Experimental Tests	ITT (Insulin Tolerance Test)	Rarely	Commonly	Assesses glucose decay post-insulin administration with limited clinical application
	CIGMA (Continuous Infusion Glucose Model Assessment)	Rarely	Commonly	Experimental and not widely validated
Simple surrogate indices	HOMA-IR/HOMA2	Commonly	Commonly	Correlates fasting glucose–insulin levels widely used in clinical settings
	QUICKI	Commonly	Commonly	Log-transformed fasting glucose–insulin correlates well with HEC
	Matsuda/OGTT-derived ISI	Limited	Commonly	Indicates hepatic and peripheral insulin sensitivity
	FGIR	Limited	Commonly	Simple fasting glucose/insulin ratio
	Fasting insulin	Limited	Commonly	Rapid surrogate measure, common clinical use
	Lipid-based indices	Limited	Commonly	Includes triglycerides/HDL-C, population-dependent
Biomarkers	Fasting insulin	Commonly	Commonly	Baseline indicator for assessing insulin resistance
	IGFBP-1	Limited	Commonly	Low levels indicate IR often utilized in research
	SHBG	Limited	Commonly	Inversely correlated with insulin resistance
	Leptin/Adiponectin ratio	Limited	Commonly	Adipokine-based marker for research purposes
	TG/HDL-C ratio	Limited	Commonly	Lipid-based surrogate marker that may be influenced by ethnicity or metabolic status

“Commonly” = suitable for that setting. “Limited or Rarely” = feasible but not broadly used. Clinical-use methods are used for routine patient assessment (HOMA-IR, QUICKI, fasting insulin). Research/epidemiology involves more complex dynamic tests or biomarker ratios necessitating modeling or specific populations. HEC: hyperinsulinemic–euglycemic clamp; FSIVGTT: frequently sampled intravenous glucose tolerance; ITT: insulin tolerance test; CIGMA: continuous infusion of glucose with model assessment; HOMA-IR/HOMA2: homeostasis model assessment; QUICKI: quantitative insulin sensitivity check index; OGTT: oral glucose tolerance test; IR: insulin resistance; ISI: insulin sensitivity glycemia index; IGFBP-1: Insulin-like Growth Factor Binding Protein 1; SHBG: Sex Hormone-Binding Globulin; TG/HDL-C: triglycerides/HDL-cholesterol.

**Table 2:** Main mechanisms of insulin resistance.

Factor or Mechanism	Effect on Insulin Resistance	Target Tissue or Organ	Principal Molecules or Pathways
Insulin receptor impairment	Decreased insulin binding, disrupted intracellular signaling	Muscle, liver, adipose tissue, kidney	INSR, IRS1/2, PI3K, AKT/PKB, PTP1B, PKC, JNK, IKK
Abnormal insulin signaling	Reduced glucose uptake and metabolic dysregulation	Muscle, liver, adipose tissue	PI3K-AKT/PKB pathway, Ras-MAPK pathway, GSK3, PDE3B
Inflammation	Cytokine-driven IRS inhibition leading to decreased insulin sensitivity	Adipose tissue, liver, skeletal muscle	TNF-α, IL-1β, IL-6, MCP-1, CRP, JNK, IKKβ, NF-κB, TLR4
Immune system activation	Stimulation of pro-inflammatory pathways and dysfunction in adipose tissue	Adipose tissue, systemic	M1 macrophages, CD4+/CD8+, T cells, B cells, NK cells, ROS
Hypoxia	Impaired glucose uptake along with inflammation and β-cell dysfunction	Adipose tissue, skeletal muscle, pancreas	HIF-1α, HIF-2α, NOX4, AMPK
Lipotoxicity	Lipid metabolites like ceramides and DAG hinder proximal insulin signaling causing mitochondrial dysfunction	Muscle, liver, adipose tissue, heart	CerS1-6, DAG, PKC isoforms, PP2A, ROS
Organelle interaction	ER stress leading to mitochondrial dysfunction and disrupted signaling	Muscle, liver, brain	ER, ROS
PTEN signaling	Negative modulation of the PI3K-AKT pathway disrupting insulin signaling	Muscle, liver, adipose tissue, pancreas	PTEN, PIP3
Vitamin D	Decreases oxidative stress while enhancing β-cell function; regulates lipid and glucose metabolism	Pancreas, muscle, liver, adipose tissue	Calcitriol, Ca^2+^ flux, ROS modulation
Minerals: Mg and Zn	Magnesium enhances insulin receptor functionality while reducing inflammation; Zinc modulates insulin secretion	Pancreas, muscle, liver, adipose tissue	Mg^2+^, Zn^2+^
Insulin actions on nervous system	Regulates feeding behavior alongside cognitive function	Hypothalamus, hippocampus	GLUT4, insulin receptors, cerebral blood flow
Autophagy	Preserves organelle functionality; regulates insulin signaling; diminishes mitochondrial stress	Muscle, liver, adipose tissue	ATG proteins, BCL2, FGF21, ATF4, ER function
Gut microbiota	Modulates inflammation, SCFA production, energy metabolism	Gut, systemic	SCFAs, BCAAs, TLR4, Prevotella copri, butyrate

Abbreviations: IRS1/2: insulin receptor substrate; DAG: diacylglycerol; PI3K: Phosphoinositide 3-kinase, SCFA: short-chain fatty acid; INSR: Insulin receptor; PTP1B: protein tyrosine phosphatase 1B; PKC: protein kinase C; JNK: c-Jun NH2-terminal kinase; IKK: IkBa kinase; Ras-MAPK: Ras–mitogen activated protein kinase; GSK3: Glycogen Synthase Kinase-3; PDE3B: Phosphodiesterase 3B; HIF: Hypoxia-Inducible Factor; NOX4: NADPH oxidase 4; AMPK: Adenosine Monophosphate-activated Protein Kinase; CerS1-6: ceramide synthase; PP2A: protein phosphatase 2A; ROS: Reactive Oxygen Species; ER: endoplasmic reticulum; PTEN: Phosphatase and Tensin homolog; GLUT4: Glucose Transporter Type 4; ATG: autophagy-related genes; BCL2: B-cell lymphoma 2; FGF21: Fibroblast Growth Factor 21; ATF4: Activating Transcription Factor 4; BCAAs: Branched-Chain Amino Acids; TLR4: Toll-like receptor 4. Adapted and summarized from Zhao X et al. 2023 [1], Petersen MC et al. 2018 [3], Shulman GI et al. 2000 [45].

**Table 3:** Dietary interventions for insulin resistance.

Dietary Pattern	Key Features	Effects on Insulin Resistance	Study Limitations
Low-carbohydrate	<45% energy from carbohydrates, increased fats and/or protein	↓ HbA1c, ↓ fasting glucose, ↓ fasting insulin ↓ HOMA-IR	Short follow-up, heterogeneous definitions, macronutrient quality often not standardized
Ketogenic	<50 g carbohydrates/day, nutritional ketosis	↓ HbA1c, ↑ insulin sensitivity	Few long-term RCTs
Mediterranean	High in fiber, MUFAs, polyphenols; complex carbohydrates	Gradual improvement in insulin sensitivity	Adherence monitoring challenging, cultural/geographic variability
Intermittent Fasting	Time-restricted eating (TRE), Alternate-day fasting (ADF), intermittent energy restriction (IER)	↓ HOMA-IR, ↓ fasting glucose, ↓ Hba1c, ↓ fasting insulin	Protocol heterogeneity, short intervention durations, small sample size
Plant-based	Predominantly plant foods, high in fiber; low in animal products; low saturated fat	↓ HOMA-IR ↓Fasting glucose, insulin, and *C*-peptide	Residual confounding in observational studies, heterogeneous definitions
Low Glycemic Index (Low GI)	Low-GI carbohydrate foods	↓ postprandial glucose and insulin ↓ HOMA-IR	Often short-term, small sample sizes, variable adherence

Abbreviations: HOMA-IR, Homeostatic Model Assessment of Insulin Resistance; TRE, Time-Restricted Eating; ADF, Alternate-day fasting; IER, Intermittent Energy Restriction; MUFA, Monounsaturated Fatty Acids; GI, Glycemic Index. Notes: ↓ indicates a decrease in the reported outcome; ↑ indicates an increase in the reported outcome. Evidence from selected major dietary intervention studies and systematic reviews on insulin resistance adapted from [Vetrani et al., 2023; Estruch et al., 2018; Chen et al., 2018; Patterson et al., 2017].

**Table 4:** Evidence on Mediterranean diet.

Authors (Year)	Population	Study Design	Study Duration	Definition	Outcome Measure	Main Result
Vetrani et al. (2023) [70]	Adults with overweight/obesity (*n* = 62)	Cross-sectional observational study	/	Adherence to the Mediterranean Diet	Insulin resistance (HOMA-IR); insulin secretion and β-cell function indices (e.g., insulinogenic index, disposition index derived from OGTT); glucose and insulin response during OGTT	High MD adherence, and in particular the consumption of fish, is associated with a decreased IR in individuals with overweight/obesity
Catalán-Lambán et al. (2023) [72]	Children and adolescents with abdominal Obesity (*n* = 122)	RCT	2 years	Moderately hypocaloric mediterranean diet vs. usual care	Metabolic: fasting insulin, HOMA-IR; Sleep parameters (latency, efficiency, WASO, awakenings)	Intervention group showed significant within-group reductions in fasting insulin and HOMA-IR at 2- and 12 months vs. baseline, but no significant differences between groups
Asoudeh et al. (2023) [73]	Adolescents with PCOS (*n* = 70)	RCT	12 weeks	MD vs. dietary advice (Food Pyramid)	HOMA-IR, fasting blood glucose, anthropometric measures, inflammatory markers, systolic and diastolic blood pressure	MD led to a significant decrease in fasting blood glucose, HOMA-IR, LDL, TGs and increase in serum levels of high-density lipoprotein (HDL). In addition, resulted in a significant reduction in serum levels of inflammatory markers
Bajerska et al. (2018) [74]	Postmenopausal women with central obesity (*n* = 144)	RCT	16 weeks	Energy-restricted Mediterranean diet (MED) moderate in fat and high proportion of MUFAs vs. energy-restricted Central European diet (CED) low in fat, moderate in carbohydrates, and high in dietary fiber	Weight, waist circumference (WC), visceral fat (VF), fasting glucose (GLU), insulin (INS), HOMA-IR, lipid profile (TC, TGs, HDL-c, LDL-c) blood pressure	Both diets induced significant weight loss, WC, VF, GLU, INS, HOMA-IR, TC, TGs and BP. Improvements in metabolic syndrome risk factors were similar for both diets with no significant differences between
Blancas-Sánchez et al. (2022) [75]	Children with prediabetes (*n* = 29)	RCT	6 weeks	Adapted Mediterranean Diet (experimental) vs. standardized healthy diet (control)	Anthropometrics (waist, arm, hip circumferences, BMI, body fat %, fat-free mass); Glycemic: HbA1c, fasting insulin	HbA1c decreased significantly in both groups, but fasting insulin decreased significantly only in MD with a significant between-group difference for insulin
Salas-Salvadó et al. (2011) [77]	Adults with cardiovascular high risk without diabetes (*n* = 418)	RCT	Median follow-up ≈ 4 years	Mediterranean diet supplemented with extra-virgin olive oil (1 L/week) or nuts (30 g/die), control low-fat diet	Incidence of type 2 diabetes diagnosed	Significant reduction in type 2 diabetes incidence in Mediterranean diet groups vs. control
Ruiz-Canela et al. (2025) [81]	Adults with metabolic syndrome and overweight or obesity (*n* = 4746)	RCT	6 years	MedDiet (planned reduction of 600 kcal per day), increased physical activity, and behavioral strategies for reducing weight, or a control group receiving ad libitum MedDiet advice	Type 2 diabetes incidence, weight, waist circumference, adherence, physical activity	Intensive intervention (energy-reduced MedDiet + physical activity + behavioral strategies) reduced type 2 diabetes incidence by ~31% vs. ad libitum MedDiet

Abbreviations: RCT, Randomized Controlled Trial, MD or MED, Mediterranean Diet, CED Centrale European diet, HOMA-IR, Homeostatic Model Assessment of Insulin Resistance, HbA1c, Glycosylated Hemoglobin; WASO (wakefulness after sleep onset); TC, Total Cholesterol; LDL-c, Low Density Lipoprotein Cholesterol; TGs, Triglycerides, MUFAs, Monounsaturated Fatty Acids; BMI, Body Mass Index, WC, Waist Circumference; VF, Visceral Fat.

**Table 5:** Evidence on DASH diet.

Authors (Year)	Population	Study Design	Study Duration	Definition	Outcome Measure	Main Result
Foroozanfard et al. (2017) [84]	Women with PCOS (*n* = 60)	RCT	12 weeks	Hypocaloric DASH diet vs. hypocaloric control diet	BMI, insulin, HOMA-IR, insulin sensitivity check index (ISI), anti-Müllerian hormone (AMH), free androgen index (FAI), sex hormone-binding globulin (SHBG), nitric oxide (NO), malondialdehyde (MDA)	Compared with control diet, the DASH diet led to greater reductions in BMI, insulin, HOMA-IR and insulin sensitivity check index.
Asemi et al. (2015) [85]	Women with PCOS (*n* = 48)	RCT	8 weeks	DASH vs. control diet	Insulin resistance (serum insulin, HOMA-IR), serum hs-CRP, waist & hip circumferences	DASH eating pattern, compared to the control diet, resulted in a significant reduction in serum insulin levels and HOMA-IR

Abbreviations: RCT, Randomized Controlled Trial; HOMA-IR, Homeostatic Model Assessment of Insulin Resistance; DASH, Dietary Approaches to Stop Hypertension; hs-CRP, High-Sensitivity *C*-Reactive Protein; PCOS, Polycystic Ovary Syndrome.

**Table 6:** Evidence on low-GI diets.

Authors (Year)	Population	Study Design	Study Duration	Definition	Outcome Measure	Main Result
Yu et al., 2025 [88]	Adults without diabetes (*n* = 192)	Meta-analysis of RCTs	7 days to 6 months	Low-GI diets ≤ 55 vs. high-GI diets ≥ 70	HOMA-IR	Low-GI diets significantly reduced HOMA-IR vs. high-GI
Solomon et al., 2010 [89]	Obese, prediabetic adults (*n* = 22)	RCT	12 weeks	Low-GI ~40 diet + exercise vs. high-GI 80 diet + exercise	HOMA-IR, insulin sensitivity (hyperinsulinemic–euglycemic clamp), oral glucose-induced insulin secretion, postprandial glucose-dependent insulinotropic polypeptide (GIP) responses, body composition	Low-glycemic index (GI) diet + exercise improved insulin resistance similarly to a high-GI diet + exercise, but only the low-GI group showed reduced postprandial hyperinsulinemia and suppressed GIP response
Khorshidi et al., 2026 [90]	Children/adolescents with overweight/obesity	Meta-analysis of RCTs	5–24 weeks	Low-GI (≤55) and reduced-carb diets vs. control	HOMA-IR, fasting insulin	Low-GI diets combined with calorie restriction significantly reduced fasting insulin and HOMA-IR, and balanced carbohydrate low-GI diets also lowered HOMA-IR; reduced-carbohydrate diets alone did not significantly affect insulin or HOMA-IR
Liu et al., 2009 [91]	Adults without diabetes (Framingham Offspring Study, *n* ≈ 2875)	Cross-sectional	1991–995	Habitual diet/dietary patterns (observational) low-GI pattern ≤ 55 vs. high-GI pattern ≥ 70.	Waist circumference, BMI, 2 h post-challenge insulin, fasting insulin, insulin sensitivity index (ISI_0_,_120_), HDL-cholesterol, TAG triacylglycerol and blood pressure	Diets high in fruits, whole grains, and reduced-fat dairy associated with lower waist circumference and BMI; diets high in refined grains, sweets, and soda associated with higher fasting insulin and less favorable insulin-resistant phenotypes; 2 h post-challenge insulin differences not significant

Abbreviations: RCT, Randomized Controlled Trial; HOMA-IR, Homeostatic Model Assessment of Insulin Resistance; Low-GI, Low Glycemic Index; High-GI, High Glycemic Index.

**Table 7:** Evidence on plant-based diets.

Authors (Year)	Population	Study Design	Study Duration	Definition	Outcome Measure	Main Result
Termannsen et al. (2024) [92]	Adults with overweight/obesity, prediabetes or T2D (*n* = 716)	Meta-analysis of RCTs	4–24 weeks	Plant-based diets (vegan/vegetarian) vs. control diets	HOMA-IR, fasting insulin	Plant-based diets improved insulin sensitivity markers compared with control diets in overweight/obesity population; HOMA-IR ↓ (*p* = 0.007); fasting insulin ↓ (*p* = 0.009)
Kahleova H. et al. (2018) [93]	Adults with overweight/obesity (*n* = 75)	RCT	16 weeks	Low-fat plant-based diet vs. control diet	β-cell function (meal test), HOMA-IR, body composition, BMI	Plant-based diet reduces fasting plasma glucose, insulin, *C*-peptide, HOMA-IR, body weight and fat visceral fat
Kahleova H. et al. (2020) [94]	Overweight adults (*n* = 244)	RCT	16 weeks	Low-fat vegan diet vs. control diet	Body weight, HOMA-IR, postprandial metabolism, intramyocellular and hepatocellular lipids	Vegan diet reduced body weight, HOMA-IR, intramyocellular and hepatocellular lipids
Chen et al. (2018) [95]	Adults from the Rotterdam Study (*n* = 6798)	Prospective cohort study	Median follow-up ≈7 years	Plant-based dietary index (range 0–92) reflecting higher plant vs. animal food intake	HOMA-IR, incidence of prediabetes and type 2 diabetes	Higher adherence to plant-based diets was associated with significantly lower insulin resistance

Abbreviations: RCT, Randomized Controlled Trial; HOMA-IR, Homeostatic Model Assessment of Insulin Resistance. Notes: ↓ indicates a decrease in the reported outcome.

**Table 8:** Evidence on low-carbohydrate diets.

Authors (Year)	Population	Intervention Study Type	Duration	Definition	Outcome Measure	Main Result
Lan et al. (2025) [98]	Adults with T2DM (*n* = 2831)	Systematic review and meta-analysis of RCTs	3–208 weeks	Low carbohydrate or very-low-carbohydrate diet vs. conventional low-fat diets	HbA1c, fasting glucose, HOMA-IR, fasting insulin, BMI	Low-carbohydrate diets improved glycemic control (↓ HbA1c, ↓ fasting glucose) and reduced insulin resistance (↓ HOMA-IR), particularly in short-term interventions (3 months)
Yuan et al. (2020) [99]	Adults with T2DM (*n* = 67)	Systematic review and meta-analysis of RCTs	Variable (1–56 weeks)	Ketogenic diet vs. control diets	HbA1c, fasting glucose, lipid profile, weight	Ketogenic diet significantly reduces fasting glucose, HbA1c and body weight
Foster et al. (2003) [100]	Adults with obesity (*n* = 63)	RCT	12 months	Low-carbohydrate diet (Atkin’s diet) vs. low-fat diet	Body weight, insulin sensitivity, metabolic parameters	No significant changes in insulin sensitivity. Greater short-term weight loss and metabolic improvements at 3 months and 6 months
Al-Reshed (2023) [101]	Normal-weight healthy adults (*n* = 120)	Cross-sectional observational study	At least 7 days	Low-carbohydrate diet (<45% of daily energy intake from carbohydrates) vs. recommended range of carbohydrate group (45–65% of daily energy intake) vs. high-carbohydrate group (> 65% of daily energy intake)	HOMA-IR, HOMA-β and *C*-peptide	Low carbohydrate intake associated with higher insulin resistance markers
Merovci et al. (2024) [102]	Adults with overweight/obesity and T2D (*n* = 29)	RCT	10 days	Standard weight-maintaining diet vs. weight-maintaining ketogenic diet vs. weight-maintaining ketogenic diet fat supplemented	FPI, FPG, *C*-peptide, HOMA-IR, HbA1c, hepatic glucose production and total body (muscle) glucose disposal	Ketogenic diet in absence of weight loss has no beneficial effect on glucose tolerance and insulin sensitivity
Sumithran (2013) [103]	Adults with overweight or obesity (*n* = 39)	Non-randomized interventional study	10 weeks	Ketogenic very-low-energy diet + maintenance diet	Appetite-related hormones (leptin, ghrelin, PYY, CCK, insulin); subjective appetite ratings, FI, FG, HOMA-IR, BHB	Weight loss led to significant reductions in FG and FI and improved HOMA-IR from week 0 to 8

Abbreviations: RCT, Randomized Controlled Trial; BMI, Body Mass Index; HbA1c, Glycosylated Hemoglobin; HOMA-IR, Homeostatic Model Assessment of Insulin Resistance; HOMA-β, Homeostatic Model Assessment beta-cell function; FPI, Fasting Plasma Insulin; FPG, Fasting Plasma Glucose; FG, Fasting Glucose; FI, Fasting Insulin; BHB, Beta-Hydroxybutyrate.

**Table 9:** Evidence on intermittent fasting.

Authors (Year)	Population	Intervention Study Type	Duration	Definition	Outcome Measure	Main Result
Paravaresh et al. (2019) [110]	Adults with metabolic syndrome (*n* = 69)	RCT	8 weeks	Alternate-day fasting vs. calorie restriction	Anthropometric parameters, blood pressure, fasting plasma glucose, fasting insulin, HOMA-IR and lipid profile	Alternate-day fasting diet significantly reduced anthropometric parameters, systolic blood pressure and fasting plasma glucose. No significant difference on HOMA-IR and fasting insulin concentration
Kunduraci et al. (2020) [111]	Adults with metabolic syndrome (*n* = 70)	RCT	12 weeks	Intermittent energy restriction (IER) intervention group and continuous energy restriction (CER) control group	Lipid profile, fasting plasma glucose, insulin, HbA1c, HOMA-IR, blood pressure and body composition	Fasting glucose and insulin decreased in both groups. No significant differences were observed between the IER and CER groups
Guo et al. (2021) [112]	Adults with metabolic syndrome (*n* = 39)	RCT	8 weeks	2-day fasting dietary schedule (IF) vs. control diet (CD)	Anthropometric parameters; lipid profiles: TC, HDL-c, LDL-c, TGs, ApoA1, ApoB; serum glucose and insulin, plasma and cytokines of systemic inflammation; adipokines: leptin and adiponectin; biomarkers of oxidative stress; biomarkers of endothelial function and gut-derived metabolites	IF did not result in statistically significant changes in glucose metabolism parameters, including fasting blood glucose, fasting insulin, and HOMA-IR, although non-significant improving trends were observed
Cramer et al. (2022) [113]	Adults with metabolic syndrome (*n* = 145)	RCT	24 weeks	5-day fasting followed by 10 weeks of lifestyle modification vs. 10 weeks of lifestyle modification only	HOMA index, insulin, glucose, HbA1c, diastolic blood pressure, anthropometric parameters, lipids profile, IL-6, CRP, IGF-1, creatinine, eGFR and acid uric	Fasting significantly reduced HOMA index, insulin and HbA1c after 1 week and glucose at week 24
Yuan et al. (2022) [114]	Adults with metabolic syndrome or diabetes (*n* = 359)	Meta-analysis of RCTs	5 weeks–12 months	Different types of intermittent fasting: IER, IECR, TRF, IF, IFCR-L, IFCR-F	Fasting glucose, insulin, HbA1c, HOMA-IR, lipid profile, anthropometric parameters	IF diets reduce fasting glucose, insulin and HOMA-IR

Abbreviations: RCT, Randomized Controlled Trial; HbA1c, Glycosylated Hemoglobin; HOMA-IR, Homeostatic Model Assessment of Insulin Resistance; IER, Intermittent Energy Restriction; IECR, Intermittent Energy Carbohydrate Restriction; IF, Intermittent Fasting; TRF, Time Restricted Feeding; IFCR-L, Intermittent Fasting Calorie Restriction-Liquid Diet; IFCR-F, Intermittent Fasting Calorie Restriction-Food based diet; TC, Total Cholesterol; HDL-c, High Density Lipoprotein Cholesterol; LDL-c, Low Density Lipoprotein Cholesterol; TGs, Triglycerides; ApoA1, Apolipoprotein A1; ApoB, Apolipoprotein B.

## Data Availability

No new data were created or analyzed in this study. This article is a review of previously published literature.

## Abbreviations

The following abbreviations are used in this manuscript:

IR	insulin resistance
T2DM	type 2 diabetes
MASLD	metabolic dysfunction-associated steatotic liver disease
RCT	randomized controlled trials
PCOS	polycystic ovary syndrome
T1DM	type 1 diabetes
TyG	triglyceride–glucose index
